# Impaired orienting function detected through eye movements in patients with temporal lobe epilepsy

**DOI:** 10.3389/fnins.2023.1290959

**Published:** 2023-12-22

**Authors:** Shirui Wen, Huangyemin Zhang, Kailing Huang, Xiaojie Wei, Ke Yang, Quan Wang, Li Feng

**Affiliations:** ^1^Department of Neurology, Zhongshan Hospital, Fudan University, Shanghai, China; ^2^Department of Neurology, Xiangya Hospital, Central South University, Changsha, Hunan, China; ^3^National Clinical Research Center for Geriatric Disorders, Xiangya Hospital, Central South University, Changsha, Hunan, China; ^4^Key Laboratory of Spectral Imaging Technology, Xi'an Institute of Optics and Precision Mechanics of Chinese Academy of Sciences, Xi'an, China; ^5^University of Chinese Academy of Sciences, Beijing, China; ^6^Key Laboratory of Biomedical Spectroscopy of Xi'an, Xi'an Institute of Optics and Precision Mechanics of Chinese Academy of Sciences, Xi'an, China

**Keywords:** orienting attention, eye-tracking technology, temporal lobe epilepsy, attention network test, visual attention

## Abstract

**Objective:**

Patients with temporal lobe epilepsy (TLE) often exhibit attention function impairment. The orienting network is the subsystem of the attention network that has not been fully studied. In this study, we used eye-tracking technology with an attention network test (ANT)-based task to assess the orienting function of TLE patients, aiming to characterize their eye movement patterns.

**Methods:**

A total of 37 TLE patients and 29 healthy controls (HCs) completed the ANT task based on eye-tracking technology. Orienting function damage was mainly assessed by the ANT orienting effect. Eye movement metrics, such as mean first goal-directed saccade latency (MGSL), total saccades, and saccade amplitudes, were compared between groups

**Results:**

The TLE patients had a significantly lower ANT orienting effect (HC, 54.05 ± 34.05; TLE, 32.29 ± 39.54) and lower eye-tracking orienting effect (HC, 116.98 ± 56.59; TLE, 86.72 ± 59.10) than those of the HCs. The larger orienting effects indicate that orienting responses are faster when receiving a spatial cue compared with a center cue. In the spatial cue condition, compared with HCs, the TLE group showed a longer first goal-directed saccade latency (HC, 76.77 ± 58.87 ms; TLE, 115.14 ± 59.15 ms), more total saccades (HC, 28.46 ± 12.30; TLE, 36.69 ± 15.13), and larger saccade amplitudes (HC, 0.75° ± 0.60°; TLE, 1.36° ± 0.89°). Furthermore, there was a positive correlation of the orienting-effect score between the ANT task and eye-tracking metrics (*r* = 0.58, *p* < 0.05).

**Conclusion:**

We innovatively developed a new detection method using eye-tracking technology in combination with an ANT-based task to detect the orienting function in TLE patients. The current research demonstrated that TLE patients had a significant orienting dysfunction with a specific saccade pattern characterized by a longer first goal-directed saccade latency, more total saccades, and larger saccade amplitudes. These oculomotor metrics are likely to be a better indicator of orienting function and may potentially be used for behavioral-based interventions and long-term cognition monitoring in TLE patients.

## 1 Introduction

Attention has been implicated as a foundation of cognitive function and can be divided into three distinct subnetworks: alerting, orienting, and executive network (Posner and Petersen, 1990; Petersen and Posner, 2012). A decline in attention has been observed in patients with epilepsy (Englot et al., 2020; Rainer et al., 2023). The literature on this topic reports that patients with temporal lobe epilepsy experience alerting and executive network impairment, which is associated with reduced white matter fibers and disrupted brain networks between temporal and frontal lobes (Cataldi et al., 2013; Tyler et al., 2015; Englot et al., 2020; Liu et al., 2020; Zhou et al., 2021). However, few studies have focused on orienting performance among TLE patients, which plays a crucial role in prioritizing information presented by the environment for further processing and maintaining target tracking, social attention, emotions, and thought regulation (Isaacowitz et al., 2006; Posner et al., 2012). Therefore, it is imperative to elucidate the orienting function in TLE patients and investigate characteristic eye movement patterns to further interrupt and raise awareness of frontal lobe-related cognitive (i.e., attention function, executive function, and higher-order cognition) impairment.

Existing assessments of orienting function mainly focus on audio and vision (Lee and Spence, 2015, 2017) and rely mostly on neuropsychological assessments, such as the Attention Network Test (ANT) (Fan et al., 2002), which could reflect the orienting function impairment to a certain extent. Rapid eye movement is considered critical for visual perception and orienting function by enhancing eye scanning behavior, but it is usually not measured in the ANT task. When the sudden appearance of a stimulus is in one direction, reactive saccades would be triggered and voluntary saccades might be intentionally driven (Everling, 2007). Additionally, previous neuroimaging and EEG studies have suggested the anatomical overlaps and interactions between eye saccadic adaptation and orienting attention systems (Corbetta, 1998; De Haan et al., 2008). Furthermore, Dragone et al. identified a correlation between alterations in the predictive cues that guide voluntary orienting attention and variations in pupil dilation while healthy participants performed the ANT task (Dragone et al., 2018). This indicates that eye tracking may have special advantages in the quantitative assessment of orienting function. Based on the above, eye tracking, as an emerging cognitive assessment that enables the recording of real-time ocular behavior during the performance of the ANT task, represents incentives to greatly improve the screening process of target focusing, smooth pursuit, and eye scanning. Such technology may provide more insight into the neurobehavioral mechanisms.

As far as we know, this is the first study to explore whether this coupling in the performance of the orienting function exists between original scores of ANT tasks and eye-tracking metrics. Moreover, we sought to demonstrate the characteristic pattern of orienting network perturbations in TLE patients and provide the possibility to probe into the neural manifestation of interactions and the principle of comorbidity in cognitive impairment accompanying TLE.

## 2 Methods

### 2.1 Participants

This study was carried out in the Department of Neurology, Xiangya Hospital of Central South University, from December 1, 2019 to December 30, 2021. In total, we recruited *N* = 37 TLE patients and *N* = 29 healthy controls (HC) ranging in age from 16 to 60 years. These patients were diagnosed with temporal lobe epilepsy according to the seizure classification and epilepsy syndrome proposed by the International League Against Epilepsy (ILAE) in 2017 (Scheffer et al., 2017). The etiology was classified on the basis of the consensus opinion of two epilepsy experts using ILAE criteria (Scheffer et al., 2017). Those with structural abnormalities related to epilepsy in other brain regions or with a family history of epilepsy were excluded at recruitment. Six patients never took any anti-epileptic drugs (AEDs) or had withdrawn from AEDs for more than 6 months. Twenty-four TLE patients received monotherapy. The remaining seven patients were taking two or more AEDs (combinations of carbamazepine, oxcarbazepine, valproate acid, levetiracetam, lamotrigine, or topiramate).

The healthy control group consisted of 29 healthy volunteers matched to the patient group in terms of age, sex, and years of education. None of the healthy control participants had a history of psychiatric or neurological disorders. All participants were right-handed with normal or corrected-to-normal hearing and vision. The demographic and clinical characteristics are summarized in Table 1. All procedures were approved by the hospital's Medical Research Ethics Committee and informed written consent was obtained before enrollment in the study.

**Table 1:** Characteristics of the participants.

	**Healthy control group (*n* = 29)**	**TLE patient group (*n* = 37)**	***P-*value**
Gender (male)	29 (16)	37 (17)	0.628
Age (mean ± SD)	32.66 ± 11.97	31.22 ± 9.83	0.593
Education (years)	14.03 ± 4.00	12.62 ± 3.25	0.119
Age at onset (years)	—	22.31 ± 10.66	
Course of epilepsy (years)	—	—	
<5 years	—	13	
5–10 years	—	12	
>10 years	—	12	
AEDs [0–1/≥2 (i.e., polytherapy)]	—	30/7	
Laterality (left/right)	—	18/19	
Etiology (structural/unknown)	—	20/17	

Based on commonly published risk factors (Hermann et al., 2006, 2010; Vinti et al., 2021; Arslan and Demir, 2022) of attention dysfunction in TLE patients, the following characteristics were collected: gender (male/female), the course of epilepsy at the time of the neuropsychology assessment (<5 years/5–10 years/>10 years), laterality of epilepsy (left/right), number of anti-epileptic drugs [AEDs; 0–1/≥2 (i.e., polytherapy)], and etiology {known cause [i.e., structural (hippocampal sclerosis, cerebrovascular disease-related)]/unknown cause (i.e., unknown or suspected as genetic)}.

### 2.2 Neurobehavioral assessment

We developed an experimental platform for automatic attention function evaluation based on the Eyelink 1000 Plus Desktop Mount eye tracker (SR Research) combined with the ANT (version Attention Exp1.1B5) via Experiment Builder (version 2.3.38). All participants completed the ANT. Eye movement tracking was performed simultaneously during the task.

**Figure 1:**
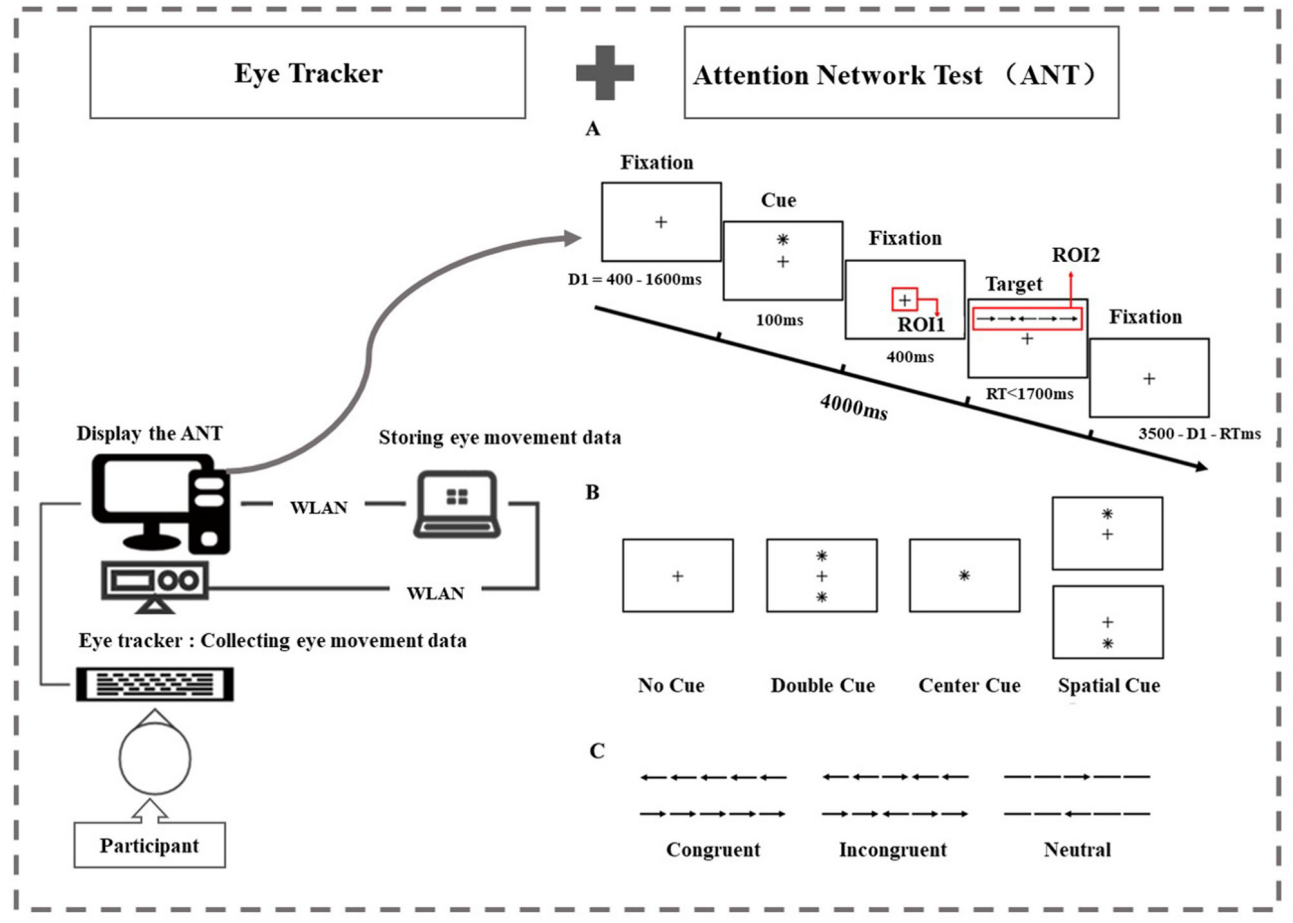
Task examples and regions of interest (ROIs). **(A)** The attention network test: an example of the procedure. There were two regions of interest. In the fixation stage, we set a square of 80 × 80 pixels centered on the fixation point “+” as ROI 1. In the target stimulation stage, we set a rectangle of 280 × 80 pixels centered on the target stimulus as ROI 2. **(B)** The four cue condition: for the no-cue trials, participants saw only a fixation for 100 msec. For the center-cue trials, participants were shown an asterisk at the location of the fixation cross. For the double-cue trials, there were two warning cues corresponding to the two possible target positions—up and down. For the spatial-cue trials, the cue was at the target position. **(C)** The three flanker conditions: the six stimuli used in the present experiment. The target stimuli were flanked on either side by two arrows in the same direction (congruent condition) or in the opposite direction (incongruent condition), or by lines (neutral condition).

In the ANT, stimuli were displayed on an LCD monitor with a screen size of 41.5 × 34.6 cm and a resolution of 1,280 × 1,024 pixels (Fan et al., 2002). Participants viewed the screen from a distance of 60 cm, and keystroke responses were collected via two input keys placed on the keyboard on the right side of the participant. Target stimuli consisted of a row of five visually presented horizontal black lines, with arrows pointing left or right, on a white background (Figure 1C). The target was the arrow in the center. This target was flanked on either side by two arrows or by lines. A single arrow or line consisted of 0.558° of visual angle and the contours of adjacent arrows or lines were separated by 0.068° of visual angle. The stimuli (one central arrow plus four flankers) consisted of a total 3.088° of visual angle.

To measure alerting and/or orienting, there were four warning conditions: no cue, center cue, double cue, and spatial cue (Figure 1B). This article is mainly related to the evaluation of the orienting function, so we focus on the center cue and spatial cue. For the center cue trials, participants were shown an asterisk at the location of the fixation cross for 100 msec. For the spatial cue trials, the cue was at the target position and the time course was the same as in the center cue task. This version included a full feedback practice block of 24 trials and three no-feedback experimental blocks containing 96 trials (4 cue conditions × 2 target positions × 2 target orientations × 3 flanking conditions × 2 repetitions). Each trial lasted for 4,000 ms and consisted of five events. Region of interest (ROI) has been applied in multiple studies related to attention tasks. We set up the following two regions of interest. In the fixation stage, we set a square of 80 × 80 pixels (2.48° × 2.48°) centered on the fixation point “+” located in center-screen as ROI 1. In the target stimulation stage, we set a rectangle of 280 × 80 (8.60° × 2.48°) pixels centered on the target stimulus as ROI 2 (Figure 1A).

Participants were instructed to identify the direction of each target arrow (left/right) while ignoring the flanks by pressing the corresponding button (“←”/“ → ”). Throughout the task, participants were asked to focus on a centrally located fixation point and respond as quickly and accurately as possible. One trial consisted of different cues and flanking conditions, and the display of the trial was pseudorandomized. The process of one trial is shown in Figure 1A. During the task, eye movements were detected using an Eyelink 1000 Plus Desktop Mount eye tracker (SR Research) at a monocular sampling rate of 500 Hz.

We calculated the orienting effects to determine the orienting network of the individual participants. Experiment Builder was implemented to control the experimental procedure and collect the mean reaction time (MRT) and accuracy data for every condition. The center cue condition indicates when a target will occur but not where it will occur (alerting). The spatial cue condition indicates where a target will occur (orienting) and when it will occur. When the cue that indicates the target's location (spatial cue) is subtracted from an alerting cue (center cue), the difference represents effects of orienting (Fan et al., 2002; Beane and Marrocco, 2004).


$$\begin{array}{c} \text{ANT-orienting-effect}=\text{MRT central cue}-\text{MRT spatial cue} \end{array}$$

### 2.3 Eye movement metrics and statistical analysis

Eye movement data were captured on predefined ROIs (Figure 1A). Eye movement indicators related to orienting attention were selected to objectively reflect the attention processing engineering of participants in ANT. We used Data Viewer (version 4.2.1) analysis software to extract eye movement metrics under the condition of center cue and spatial cue, including mean first goal-directed saccade latency (MGSL), total saccades, and saccade amplitudes. The calculation of MGSL, saccades, and saccade amplitudes is directly calculated by the eye-link system. MGSL was defined in all trials as the time from the targeted arrow stimulation onset to the first saccade at ROI 2, and this measure reflects the participant's alerting and orienting function, i.e., they can remain sensitive to receiving incoming information and are ready to respond. Saccade was defined as the behavior in which the acceleration of eye movements is kept at a larger level in continuous time. Total saccades are the average total saccades made during the target stimulation stage across all trials in one cue condition (e.g., spatial cue or center cue, with each cue condition containing 24 trials). Saccade amplitude (degrees) was computed as the eye displacement between saccade onset and offset. The total saccade amplitudes are the sum of saccade amplitudes in each trial. The mean saccade amplitudes are calculated by dividing the sum of the total saccade amplitudes by the total saccades in all trials under a cue condition (e.g., spatial cue or center cue, with each cue condition containing 24 trials). We chose total saccade amplitudes and mean saccade amplitudes as a couple of the eye movement metrics during the target stimulation stage. We used MGSL as a proxy for eye movement metrics to evaluate the effect of orienting:


$$\begin{array}{c} \text{Eye-tracking orienting effect}=\text{MGSL central cue}\\ -\text{MGSL spatial cue} \end{array}$$

Statistical analyses were performed using Python (Python version 3.8.3), IBM SPSS Statistics for Windows (Version 21.0.0), and the R Programming Language (version 4.0.5). Two-tailed *p*-values were calculated for all tests, with *p* < *0.05* considered statistically significant for differences. Characteristic information of participants was compared using chi-square tests for categorical variables and the independent samples *t*-test for continuous variables between TLE patients and healthy controls. In the analysis of the attention network test metrics and eye movement metrics, extreme outliers were removed and normalized. The independent samples *t*-test was then performed. The subgroup variables were TLE patients and healthy controls. The Pearson correlation coefficient was then used to analyze the association between the indicators of interest, followed by Bonferroni correction. Chi-squared tests, with *p* < 0.05 considered significant, were used to assess intergroup differences in proportions for gender, etiology, the number of medications, the course of epilepsy, and the laterality of the epilepsy, followed by Bonferroni correction, and identify univariable risk factors for orienting impairment in TLE patients.

## 3 Results

### 3.1 Demographics and clinical characteristics

There were 37 TLE patients (17 males) and 29 healthy controls (16 males) involved in this study. There were no significant differences in the demographic characteristics of both groups, as demonstrated in Table 1. There were no intergroup differences in proportions for gender, etiology, the number of medications, the course of epilepsy, and the laterality of epilepsy in the TLE group. There were no intergroup differences in orienting impairments among TLE patients for risk factors (e.g., etiology, the number of medications, the course of epilepsy, and the laterality of epilepsy) (Supplementary material 1).

### 3.2 Disrupted orienting attention in patients with temporal lobe epilepsy

**Figure 2:**
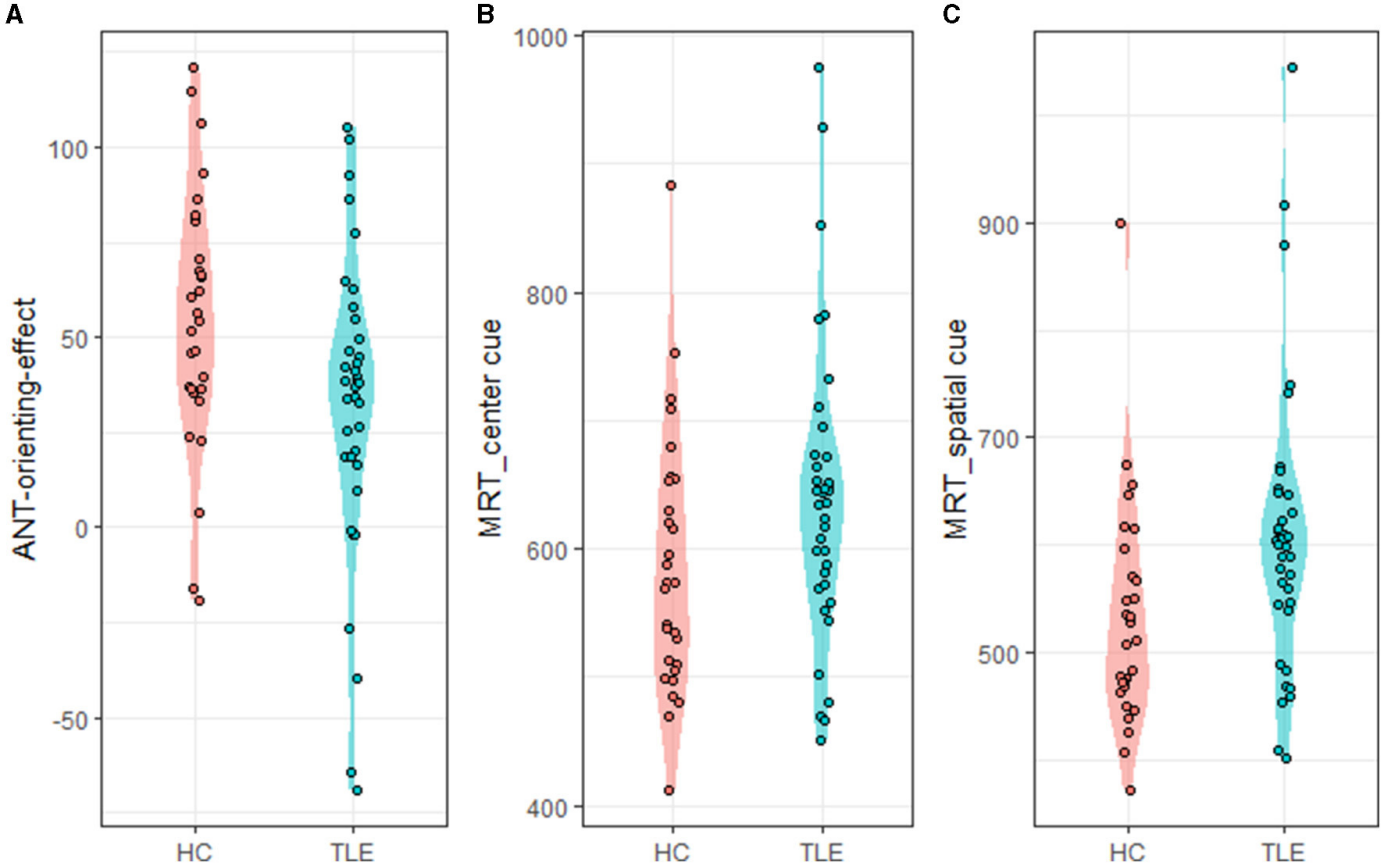
ANT-orienting-network-related metrics. ANT, attention network test; MRT, mean reaction time. **(A)** ANT-orienting-effect, **(B)** MRT_center_cue: MRT under center cue condition, and **(C)** MRT_spatial_cue: MRT under spatial cue condition.

Compared with the HC group, the TLE group had a lower ANT orienting effect (*t* = −2.40, *p* = 0.019, Cohen's *d* = 0.584), indicating that they were less helped by the orienting cue. In the center cue conditions, the TLE group showed longer RTs than the HC group (*t* = 1.997, *p* = 0.05, Cohen's *d* = 0.487). In the spatial cue conditions, the TLE group exhibited longer RTs than the HC group (*t* = 2.582, *p* = 0.012, Cohen's *d* = 0.623) (Table 2, Figure 2).

**Table 2:** Patient and control group ANT indices and eye movement metrics.

	**Healthy control group (*n* = 29)**	**TLE patients group (*n* = 37)**	***t-*value**	***p-*value**	**Cohen's *d***
ANT-orienting-effect	54.05 ± 34.05	32.29 ± 39.54	−2.400	0.019^*^	0.584
MRT (center cue)	585.80 ± 100.84	639.17 ± 115.98	1.997	0.050^*^	0.487
MRT (spatial cue)	531.75 ± 104.47	606.88 ± 131.92	2.582	0.012^*^	0.623
Eye-tracking orienting effect	116.98 ± 56.59	86.72 ± 59.10	−2.114	0.039^*^	0.522
MGSL (center cue)	193.75 ± 25.84	201.86 ± 31.40	1.151	0.250	0.279
MGSL (spatial cue)	76.77 ± 58.87	115.14 ± 59.15	2.621	0.011^*^	0.650
**Under center cue condition**					
Total saccades	38.51 ± 9.15	42.28 ± 11.24	1.468	0.147	0.364
Total saccade amplitudes (degrees)	2.10 ± 0.34	2.17 ± 0.38	0.788	0.434	0.193
Mean saccade amplitudes	1.92 ± 0.37	1.92 ± 0.33	0.32	0.975	0.008
**Under spatial cue condition**					
Total saccades	28.46 ± 12.30	36.69 ± 15.13	2.376	0.0018^**^	0.589
Total saccade amplitudes	0.75 ± 0.60	1.36 ± 0.89	3.166	0.0015^**^	0.785
Mean saccade amplitudes	0.68 ± 0.56	1.16 ± 0.69	3.094	0.0029^**^	0.749

MRT, mean reaction time; MGSL, mean first goal-directed saccade latency.

* *p* < 0.05;

** *p* < 0.01.

### 3.3 Abnormal eye movement metrics in patients with temporal lobe epilepsy

**Figure 3:**
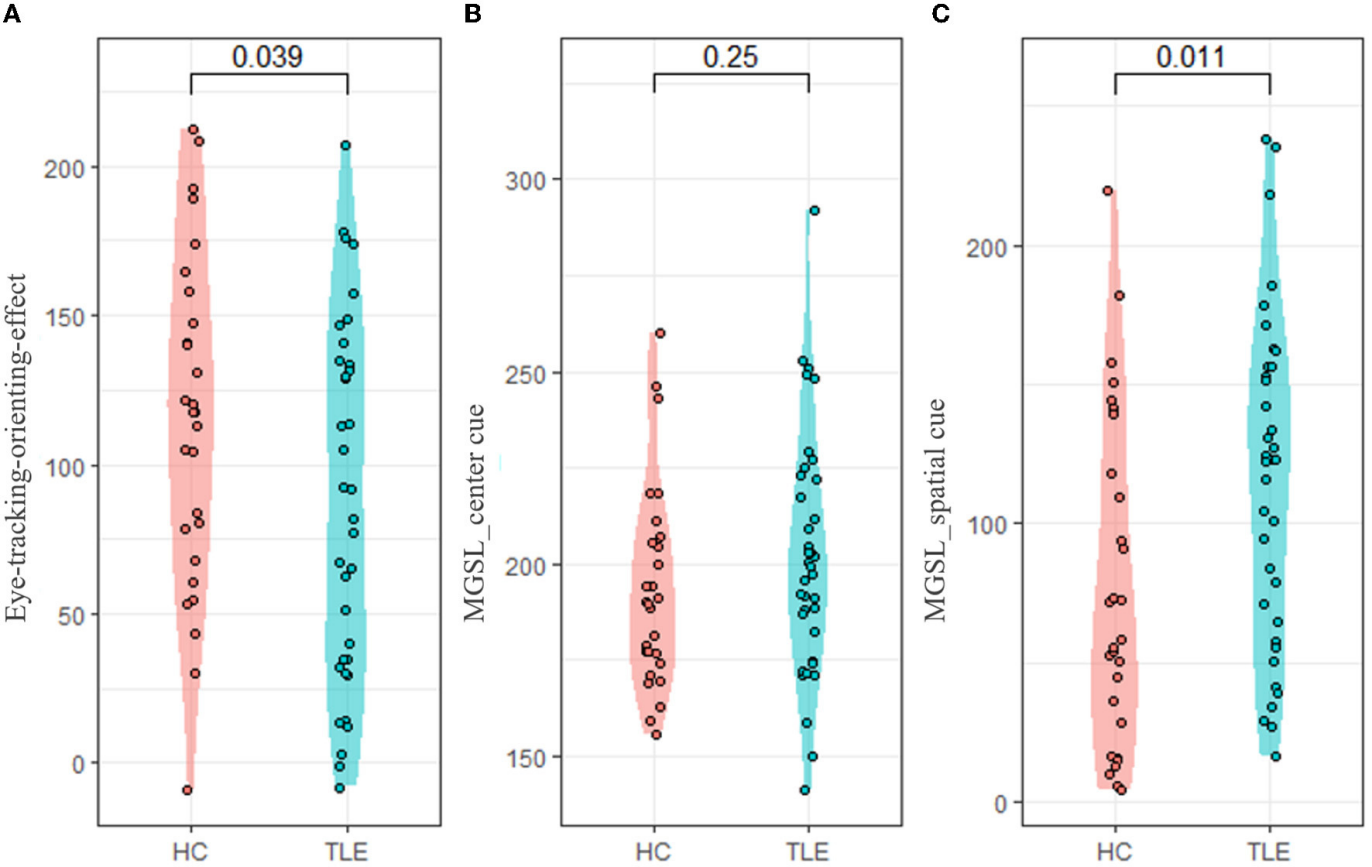
Eye-tracking-orienting-network-related metrics. ANT, attention network test; MGSL, mean first goal-directed saccade latency. **(A)** Eye-tracking-orienting-effect, **(B)** MGSL_center_cue: MGSL under center cue condition, and **(C)** MGSL _spatial_cue: MGSL under spatial cue condition.

Compared with the HC group, the TLE group had a lower eye-tracking orienting effect (*t* = −2.114, *p* = 0.039, Cohen's *d* = 0.522), representing that they benefitted less from the orienting cue. In the spatial cue conditions, the TLE group had a longer MGSL than the HC group (*t* = 2.621, *p* = 0.011, Cohen's *d* = 0.650) (Table 2, Figure 3).

**Figure 4:**
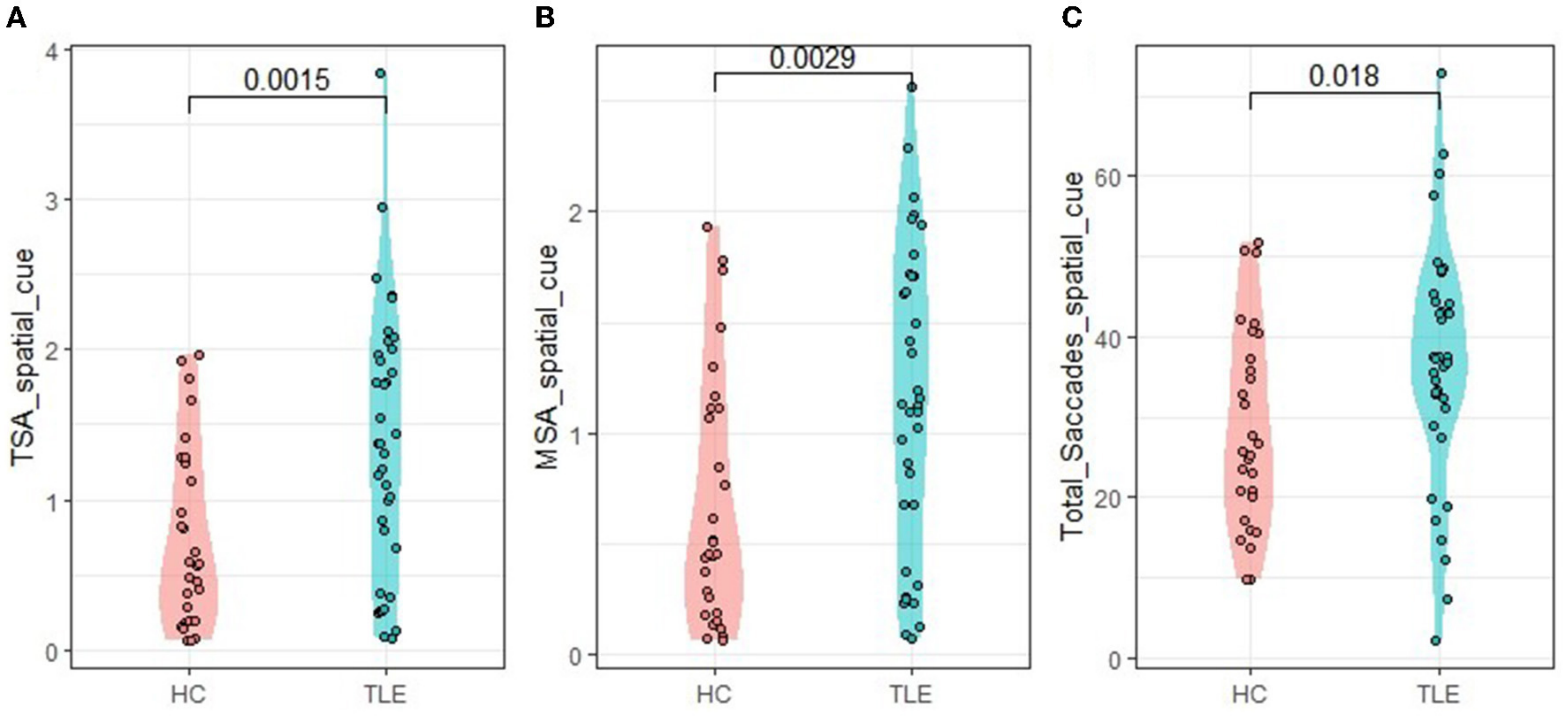
Eye-tracking metrics under the spatial cue condition. TSA, total saccade amplitudes; MSA, mean saccade amplitudes. **(A)** TSA_spatial_cue: TSA under spatial cue condition, **(B)** MSA_spatial_cue: MSA under spatial cue condition, and **(C)** Total—_Saccades-_spatial_cue: total—saccades under spatial cue condition.

We further calculated the saccade trajectories of the participants. Under the condition of center cue, there were no significant differences between the TLE and HC groups in the total saccades, total saccade amplitudes, and mean saccade amplitudes. Under the condition of spatial cue, the TLE group exhibited more total saccades than the HC group (*t* = 2.376, *p* = 0.0018, Cohen's *d* = 0.589). The TLE group showed larger saccade amplitudes (total saccade amplitudes and mean saccade amplitudes) than the HC group (*t* = 3.166, *p* = 0.0015, Cohen's *d* = 0.785; *t* = 3.094, *p* = 0.0029, Cohen's *d* = 0.749; respectively) (Table 2, Figure 4). The higher numbers of total saccades and larger saccade amplitudes suggest that, compared with healthy controls, TLE patients are relatively inaccurate in tracking a target.

### 3.4 Correlation analysis

**Figure 5:**
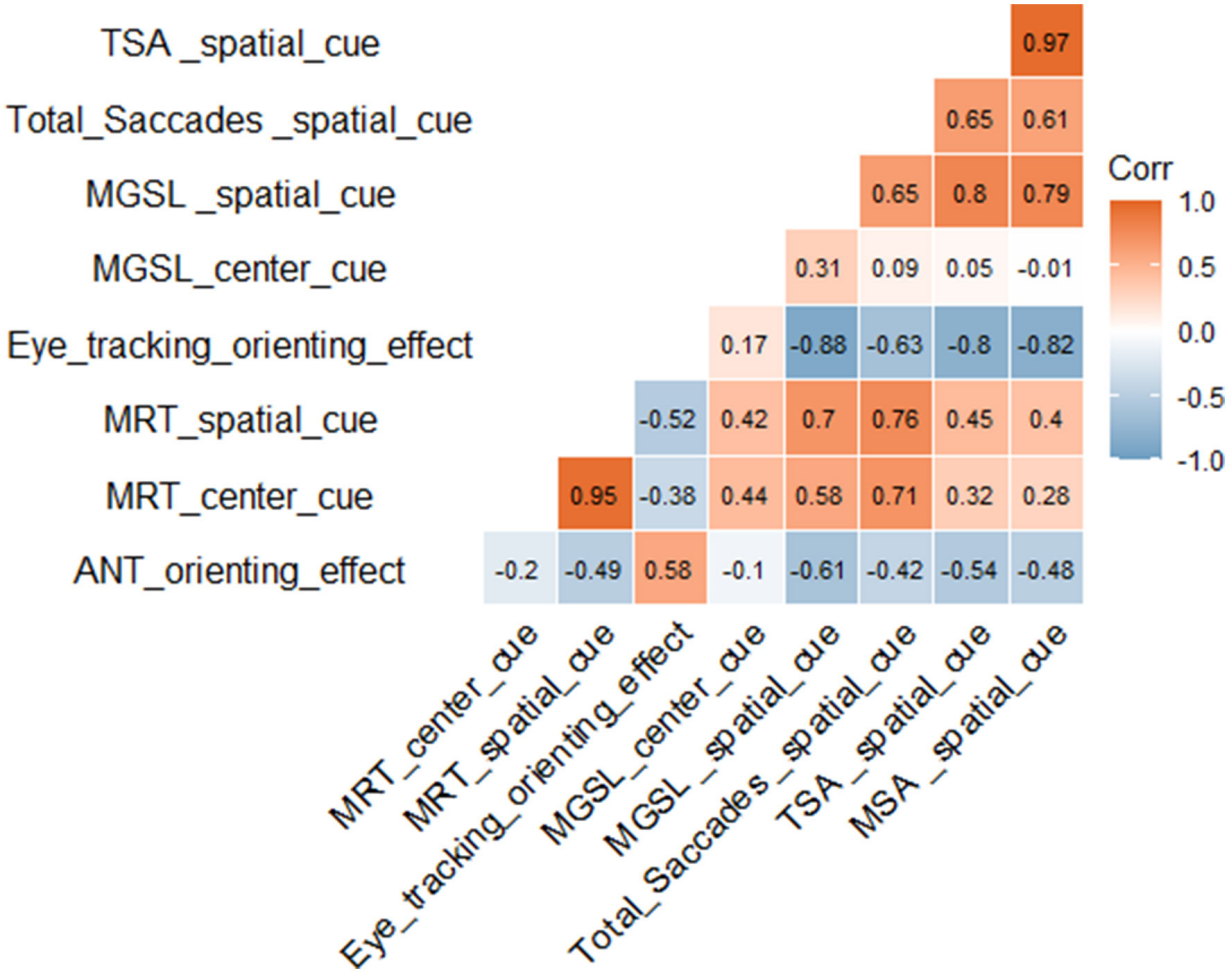
Correlation analysis. MRT, mean reaction time; MGSL, mean first goal-directed saccade latency; TSA, total saccade amplitudes; MSA, mean saccade amplitudes. Red represents a positive correlation and blue represents a negative correlation. The darker the color, the larger the correlation coefficient and the stronger the correlation.

We analyzed the correlation of participant eye movement data with the ANT data. The results showed that the eye-tracking orienting effect was significantly positively correlated with the ANT orienting effect [*r* (66) = 0.58]. Under center cue and spatial cue conditions, the first goal-directed saccade latency was positively correlated with the ANT task reaction time, [*r* (66) = 0.44 and *r* (66) = 0.70, respectively]. The eye-tracking orienting effect was significantly correlated with the total saccades [*r* (66) = −0.63, *p* < 1 × 10 – 4], total saccade amplitudes [*r* (66) = −0.802, *p* < 1 × 10 – 4], and mean saccade amplitudes [*r* (66) = −0.825, *p* < 1 × 10 – 4] (Figure 5).

## 4 Discussion

We creatively combined eye-tracking technology with the ANT to detect the attention function in TLE patients. As with previous findings (Nau et al., 2018; Qin et al., 2020; Liang et al., 2021), we found that TLE patients exhibited orienting function deficits. Even with the assistance of spatial cues, they performed worse than healthy controls in ANT tasks. Furthermore, a specific saccade pattern characterized by a longer goal-directed saccade latency, more total saccades, and larger saccade amplitudes was identified among patients with temporal lobe epilepsy during the orient attention process.

The ANT task is a neuropsychological assessment tool widely used to evaluate subnetwork deficits of attention to a certain extent (Fan et al., 2002). Visual attention is a pervasive behavior that fundamentally determines the information available for orienting (Hietanen et al., 2008; Brignani et al., 2009). However, it cannot be accurately measured due to a lack of control for viewing behavior assessment. On the contrary, eye-tracking technology can record detailed visual processes and quantify attention parameters. In combination with the ANT, eye tracking could allow us to separate the visual impact out of attention impairment. In our study, we found that TLE patients suffered from obvious orienting disturbances, with a lower eye-tracking orienting effect and worse performance in the spatial cue trials. In center cue trials, there was no significant difference between the two groups, revealing orienting dysfunction might be the most important influence on attention behavior among TLE patients. The frontal eye fields (FEFs) and the interparietal sulcus, the two essential brain regions of the orienting network, have been recognized as rapid strategic controls of attention (Corbetta and Shulman, 2002). Seizures originating from the temporal lobe usually propagate to the frontal lobes, resulting in remote abnormalities of anatomy and function. In MRI studies, TLE patients showed gray or white matter atrophy, reduced white matter fibers, and disrupted brain networks between the temporal and frontal lobes (Yang et al., 2018; Barnett et al., 2019; Leisman and Melillo, 2022; Long et al., 2022). Additionally, studies with resting-state fMRI confirmed that impaired attention function is associated with reduced functional connectivity involving the frontal cortex in TLE patients (Liu et al., 2020; Roger et al., 2020; Li et al., 2022). Therefore, it is plausible that temporal lobe epilepsy is likely to affect frontal lobes, resulting in frontal-related orienting abnormalities in TLE patients.

In our study, TLE patients presented with deficits in processing speed and accuracy compared with healthy controls. They showed more total saccades, longer first goal-directed saccade latency, and larger saccade amplitudes when trying to track visual-guided targets. It seems like the poorer orienting function in TLE patients was associated with worse oculomotor configurations toward the spatial cue task. But how can an eye movement index accurately reflect the function of spatial orienting? In terms of anatomy, the brain regions responsible for orienting function and eye movement are mostly overlapped in the frontal and parietal lobes and interact with each other. Single-unit physiology studies in the macaque have shown their important connection at the level of cell populations. Some of the cells in FEFs are active during saccades, and a distinct but overlapping population of cells are involved in the orienting function (Davidson and Marrocco, 2000). These physiological experiments provided evidence that the maintenance of orienting attention function needs the concomitant engagement of the motor system governing saccades. Therefore, the saccade and other eye movement metrics are likely to be better indicators of orienting function and can potentially be used for long-term cognition monitoring in TLE patients.

Our findings demonstrated that the eye-tracking orienting effect was significantly positively correlated with the ANT orienting effect among patients with temporal lobe epilepsy. The first goal-directed saccade latency to ROI 2 was significantly and positively correlated with the reaction time (RT) on the ANT. In addition, we found that eye movement metrics might be a potentially applicable marker for orienting function detection in TLE patients. Considering that oculomotor and orienting function are closely correlated, we advise trying to conduct orientation behavior training based on real-time eye-gaze feedback for TLE patients with an orienting deficit. With these behavioral-based interventions, we expect that the attention prognosis of TLE patients would be much improved (Kim et al., 2020).

The study had some potential limitations, but it still laid the groundwork for additional important future studies. First, the sample size was relatively small, which may indicate a potential selection bias and influence the statistical efficacy to some extent. Second, the effect of anti-epileptic drugs on eye movement metrics is a potential and unavoidable nuisance, which somewhat limits the statistical power. A multicenter large-sample prospective study is urgently needed to further validate these conclusions.

## 5 Conclusion

We innovatively established a new assay in combination with the ANT and eye-tracking technology to assess attention function among patients with temporal lobe epilepsy. TLE patients had significant orienting dysfunction with a specific saccade pattern characterized by a longer goal-directed saccade latency, more total saccades, and larger saccade amplitudes. These oculomotor metrics are likely to be a better indicator of orienting function and may potentially be used for behavioral-based interventions and long-term cognition monitoring in TLE patients. We believe that our study can promote future research on the fundamental mechanisms underlying cognitive impairment in TLE patients and provide an additional impetus to develop more effective and targeted cognitive behavioral interventions.

## Data availability statement

The original contributions presented in the study are included in the article/Supplementary material, further inquiries can be directed to the corresponding authors.

## Ethics statement

The studies involving humans were approved by Xiangya Hospital's Medical Research Ethics Committe. The studies were conducted in accordance with the local legislation and institutional requirements. Written informed consent for participation in this study was provided by the participants' legal guardians/next of kin. The subjects' consent was obtained according to the Declaration of Helsinki and approved by the Ethical Committee of the institution in which the work was performed.

## Author contributions

SW: Conceptualization, Data curation, Formal analysis, Investigation, Software, Validation, Visualization, Writing—original draft. HZ: Formal analysis, Methodology, Validation, Visualization, Writing—original draft. KH: Data curation, Investigation, Writing—original draft. XW: Methodology, Software, Validation, Writing—original draft. KY: Conceptualization, Resources, Writing—original draft. QW: Data curation, Funding acquisition, Project administration, Supervision, Writing—review & editing. LF: Funding acquisition, Project administration, Resources, Supervision, Writing—review & editing.
