# Supplementary material for: Impaired orienting function detected through eye movements in patients with temporal lobe epilepsy
Source: Front Neurosci. 2023 Dec 22;17:1290959. doi: 10.3389/fnins.2023.1290959 (PMC10770870; doi:10.3389/fnins.2023.1290959)
Supplement: Supplementary file 1 [file Table_1.DOCX]

Table 3.1 Intergroup comparison in the TLE group.

|  | TLE patients group（n=37） | | Chi-square tests (Bonferroni) | |  |
| --- | --- | --- | --- | --- | --- |
|  | **Laterality**  **(Left)** | **Laterality**  **(Right)** | |  | |
| **Course of epilepsy** (years) |  |  | | 0.826 | |
| <5 years | 7 | 6 | |  | |
| 5-10 years | 6 | 6 | |  | |
| >10 years | 5 | 7 | |  | |
| **AEDs** |  |  | | 0.734 | |
| 0-1 | 15 | 15 | |  | |
| ≥2[i.e. polytherapy] | 3 | 4 | |  | |
| **Etiology** |  |  | | 0.402 | |
| structural | 11 | 9 | |  | |
| unknown | 7 | 10 | |  | |

Note: AEDs, Anti-epileptic Drugs.

Table 3.2 Intergroup comparison of outcome measures in the TLE group (**Laterality)**

| TLE patients group（n=37） | **Laterality**  **(Left)** | **Laterality**  **(Right)** | t value | p value |
| --- | --- | --- | --- | --- |
| ANT-orienting-effect | 34.43±39.58 | 30.26±40.47 | 0.317 | 0.753 |
| Eye-tracking-orienting-effect | 86.75±65.80 | 86.68±53.81 | 0.003 | 0.997 |
| **under spatial cue condition** | | | | |
| Total saccades | 34.29±16.59 | 38.95±13.66 | -0.928 | 0.360 |
| Total saccade amplitudes | 1.35±0.89 | 1.36±0.91 | -0.045 | 0.965 |
| Mean saccade amplitudes | 1.12±0.69 | 1.19±0.69 | -0.314 | 0.755 |

Table 3.3 Intergroup comparison of outcome measures in the TLE group (Anti-epileptic Drugs, AEDs)

| TLE patients group（n=37） | **AEDs** (0-1) | **AEDs(**≥2[i.e. polytherapy]) | t value | p value |
| --- | --- | --- | --- | --- |
| ANT-orienting-effect | 29.51±33.09 | 44.18±62.31 | -0.603 | 0.566 |
| Eye-tracking-orienting-effect | 84.88±59.47 | 94.58±61.46 | -0.378 | 0.714 |
| **under spatial cue condition** | | | | |
| Total saccades | 36.66±13.14 | 36.82±23.22 | -0.018 | 0.986 |
| Total saccade amplitudes | 1.45±0.90 | 0.97±0.78 | 1.417 | 0.187 |
| Mean saccade amplitudes | 1.23±0.68 | 0.84±0.68 | 1.329 | 0.216 |

Table 3.4 Intergroup comparison of outcome measures in the TLE group **(Course of epilepsy)**

| TLE patients group | <5 years | 5-10 years | >10 years | F | p value |  |  |  |  |
| --- | --- | --- | --- | --- | --- | --- | --- | --- | --- |
| ANT-orienting-effect | 26.75±37.20 | 31.41±38.45 | 39.16±45.15 | 0.299 | 0.743 |  |  |  |  |
| Eye-tracking-orienting-effect | 76.29±62.86 | 95.25±52.57 | 89.47±64.34 | 0.327 | 0.723 |  |  |  |  |
| **under spatial cue condition** | | | | | | |  |  | 635.97±179.86 |
| Total saccades | 35.15±13.64 | 37.15±13.77 | 36.68±15.13 | 0.104 | 0.901 |  |  |  |  |
| Total saccade amplitudes | 1.39±0.77 | 1.38±0.73 | 1.28±1.17 | 0.054 | 0.947 |  |  |  |  |
| Mean saccade amplitudes | 1.21±0.68 | 1.05±0.85 | 1.15±0.68 | 0.194 | 0.825 |  |  |  |  |

Table 3.5 Intergroup comparison of outcome measures in the TLE group **(Etiology)**

| TLE patients group（n=37） | structural | unknown | t value | p value |
| --- | --- | --- | --- | --- |
| ANT-orienting-effect | 41.99±29.33 | 24.03±45.62 | 1.445 | 0.158 |
| Eye-tracking-orienting-effect | 107.42±52.36 | 69.11±59.99 | 2.050 | 0.048 |
| **under spatial cue condition** | | | | |
| Total saccades | 30.14±14.88 | 42.25±13.28 | -2.592 | 0.014 |
| Total saccade amplitudes | 1.09±0.73 | 1.57±0.97 | -1.729 | 0.093 |
| Mean saccade amplitudes | 0.97±0.64 | 1.31±0.70 | -1.512 | 0.140 |
